# ASSOCIATIONS BETWEEN COGNITIVE RESOURCES AND EMOTION REGULATION TACTICS IN AN ADULT LIFESPAN SAMPLE

**DOI:** 10.1093/geroni/igad104.0982

**Published:** 2023-12-21

**Authors:** Hannah Wolfe, Marissa DiGirolamo, Derek Isaacowitz

**Affiliations:** Northeastern University, Boston, Massachusetts, United States; Northeastern University, Boston, Massachusetts, United States; Northeastern University, Boston, Massachusetts, United States

## Abstract

The current study investigated how trait-level cognitive capacity relates to emotion regulation tactic preferences in everyday life in adulthood and old age. 51 younger adults (ages 18-39), 53 middle-aged adults (ages 40-59), and 55 older adults (ages 60+) completed measures of working memory and verbal fluency, as well as 21 days of experience sampling. On each survey, participants indicated if they had regulated since the last survey and if so, what emotion regulation strategies they used and how they implemented that strategy through specific emotion regulation tactics. Each strategy laid out by the process model (situation selection, situation modification, attentional deployment, reappraisal, response modulation) could be implemented in three specific ways (called “tactics”): positivity-upregulating, negativity-downregulating, or negativity-upregulating. For example, a person may indicate they used situation selection and then would be asked whether they chose to (a) seek out a positive situation, (b) leave a negative situation, and/or (c) enter a negative situation. Acceptance was also included as a fourth tactic type but was not categorized under any strategy. Proportions of tactics used in each instance was calculated. Acceptance use was significantly negatively correlated with working memory performance; however, this association appeared to be driven primarily by middle-aged adults. Negativity-downregulation was positively correlated with numerous cognitive capacity measures in middle-aged adults. In sum, individual differences in cognitive resources appear to play the strongest role in tactic preferences in midlife rather than old age.

